# Human papillomavirus vaccine uptake and associated factors among adolescent girls in Bona district, Sidama regional state, Ethiopia: a community-based study design

**DOI:** 10.3389/fpubh.2025.1545171

**Published:** 2025-06-27

**Authors:** Getahun Tiruye, Aster Sodo, Abera Kenay Tura, Anteneh Dirar, Adera Debella, Kasiye Shiferaw

**Affiliations:** ^1^Department of Midwifery, Hamlin College of Midwives, Addis Ababa, Ethiopia; ^2^College of Health and Medical Science, Haramaya University, Harar, Ethiopia; ^3^Institute of Health, Jima University, Jimma, Ethiopia

**Keywords:** HPV, vaccination uptake, adolescent girls, Bona district, Ethiopia

## Abstract

**Introduction:**

In developing nations, adult women’s cancer deaths are mostly caused by cervical cancer. Vaccination against the human papillomavirus (HPV) is one of the cost-effective global strategies for cervical cancer prevention, though vaccine uptake remains low in low-resource settings like Ethiopia. Despite the vaccine’s proven effectiveness in tackling cervical-related deaths, there is a dearth of evidence in Ethiopia, particularly in the study region, regarding the HPV vaccine uptake and its influencing factors.

**Objective:**

This study aimed to determine HPV vaccination uptake and its associated factors among adolescent girls aged 14–19 years in the Bona district of Sidama regional State of Ethiopia.

**Methods:**

A community-based cross-sectional study was conducted in the Bona district, Sidama region, Ethiopia, from June 1, 2024, to July 29, 2024. A systematic random sampling method was employed to recruit 833 study participants. Data were collected using a pretested, structured interviewer-administered questionnaire. The collected data were entered into Epi Data version 4.6 and exported to SPSS version 25 software for final analysis. Binary logistic regression models were used to identify factors associated with HPV vaccine uptake. Variables with a *p*-value <0.05 in the multivariable logistic regression were declared statistically significant predictors of HPV uptake.

**Results:**

In this study, the overall prevalence of HPV vaccination uptake was 49.58% [(95% CI: 46.18–52.98)]. Urban residence [AOR = 2.84 (95% CI: 1.87–4.31)], Educational status with college and above [AOR = 1.79 (95% CI 1.23–3.67)], Overall knowledge about HPV infection vaccine and cervical cancer [AOR = 2.53 (1.82–3.51)] and positive attitude towards vaccination [AOR = 2.12 (95% CI: 1.53–2.94)] were significantly associated with HPV vaccine uptake.

**Conclusion:**

Almost one in two girls in the district took the HPV vaccine. The study implies that empowering women through education, promoting health awareness about HPV, cervical cancer, and the HPV vaccine, and implementing targeted interventions for rural populations are essential means to increase HPV vaccine uptake.

## Introduction

Human Papilloma-Virus (HPV) is the most common viral infection that impacts the female reproductive system and primarily results in cervical precancerous lesions (1, 2). These lesions, which arise as a result of persistent HPV infection, are linked to 99% of cervical cancer cases (3, 4). Cervical cancer is the fourth most common cancer in women, with approximately 660,000 new cases reported in 2022. Low and middle-income countries account for an estimated 94% of cervical cancer deaths, and the regions bear the greatest disease burden due to limited access to public health services and a lack of widespread screening and treatment approaches (5).

The highest rates of both cervical cancer incidence and mortality occur in sub-Saharan Africa (SSA), Central America, and Southeast Asia (6). Eastern Africa region, where Ethiopia is also located, is one of the high-risk areas with cervical cancer rates exceeding 30 per 100,000 (7). In Ethiopia, cervical cancer is the second most prevalent and primary cause of cancer in women, claiming around 5,338 lives each year (8).

In an attempt to reduce the high toll of morbidity and mortality associated with cervical cancer-related deaths, the World Health Organization (WHO) launched HPV vaccine initiatives, urging all countries to include HPV vaccines within their national immunization programs (5). The introduction of these vaccines has demonstrated promising results in decreasing HPV infection-related diseases, including warts and precancerous lesions (2). Reports from multiple studies indicate a reduced HPV prevalence of 73–85% and a 41–57% decline in high-grade cervical lesions from countries with high coverage of HPV vaccination (9–11). While more than 90% of malignancies brought on by HPV infections could be avoided with HPV vaccination, there are significant disparities in access to immunization, with 79% coverage in high-income countries compared to just 29% in low-income countries (12).

The HPV vaccination has not been widely used in SSA, although 90% of cases of cervical cancer occur in low and middle-income countries, especially in SSA (13). In 2022, only 33% of teenage girls in 16 African nations with HPV vaccination programs had received the immunization (14). Vaccine costs, lack of funding and political support, and inadequate infrastructure for vaccine storage and transportation remain the key barriers to HPV vaccination in the region (15).

WHO announced a global strategy to eradicate cervical cancer and advises all nations to vaccinate 90% of girls by the age of 15, screen 70% of women with a high-power test by the age of 35 and ensure 90% of women with cervical disease receive treatment (16). However, HPV vaccination is still included in only 25% of low-income nations’ national programs and evidence indicated that both vaccine introduction and coverages achieved are still sub-optimal (17–19).

Since 2018, Ethiopia, with the support of the Global Alliance for Vaccine and Immunisation, has introduced the HPV vaccine for all 14-year-old girls through health centers and a school-based approach. Although an estimated two million girls have been immunized, unsubstantiated rumors about side effects or adverse outcomes that are not related to the vaccine negatively impacted public trust and have led to mistrust of the HPV vaccination program (20–22). Hence, the vaccination coverage rate remains a concern for the country, as it lags in achieving the sustainable development (SDG) targets, aiming at 90% of adolescent girls’ HPV vaccination (23).

Institutional-based studies conducted in Ethiopia showed that the percentage of teenagers who receive HPV vaccine was 45.3% in Bahir Dar City (24), 50.4% in Arba Minch (25), 66.5% in Minjar Shenkora of North Shoa (26), and 44.4% in Ambo town (27). Nevertheless, the HPV vaccination coverage report from these studies is based on a school-based approach and might not accurately reflect the true extent of the HPV vaccine rate at the community level. Therefore, community-based studies are crucial to address the limitations of these previous studies and provide a more comprehensive understanding of vaccine coverage.

To strengthen the universal coverage of the vaccine, Ethiopia has established methods addressing both supply-side and demand-side barriers, such as ensuring availability and affordability, overcoming vaccine reluctance, and boosting vaccine confidence (28). These efforts target community-based outreach initiatives in addition to school-based approaches (29). However, community-based studies that assessed HPV vaccination uptake and associated factors are very scarce in the country, particularly in the Sidama Zone, Bona district. Therefore, this study aimed to determine HPV vaccination uptake and its associated factors among adolescent girls aged 14–19 years in the Bona district of Sidama regional State of Ethiopia.

## Materials and methods

### Study area

The study was conducted in the Bona district, which is located in the eastern zone of the Sidama region at a distance of 395 km from the capital city, Addis Ababa. The district has 25 kebeles with a total population of 165,403, of which 84,356 were females and 81,047 were males. There is one general public hospital, 5 health centers and 24 health posts providing health services in the district. According to data obtained from the Bona district health office, the total number of female adolescents aged 14–19 years who lived in the district in the academic year of 2023 was 6,768.

### Study design and period

A Community-based cross-sectional study was employed from June 1 to July 29, 2024.

### Source population

All adolescent girls aged 14 to 19 years and living in the Bona district of Sidama regional state of Ethiopia were used as a source population.

### Study population

All adolescent girls aged 14 to 19 years living in the randomly selected kebeles of the Bona district of Sidama regional state, Ethiopia. Participants who were severely ill and unable to respond appropriately were excluded from this study.

### Sample size determination

The sample size for this study was determined using a single proportion population formula by considering the proportion of HPV vaccine uptake from a previously conducted community-based study in southwestern Ethiopia, (*p* = 48%) (30), 95% CI, 80% power of the test, 5% margin of error.


$$n=\frac{{\left(Z(1-\frac{\alpha }{2})\right)}^{2}\times p\times (1-P)}{d^{2}}=\frac{\left(1.96^{2})\times (0.48)(1-0.48\right)}{0.05^{2}}\approx 384.$$

However, since this study was community-based and we used a multistage sampling method, i.e., some stages to reach the study participants, we multiplied with a design effect of 2 to increase the sample size and to reduce the probability of random error (422*2 = 844). Therefore,the final sample size was determined to be 844 adolescent girls.

### Sampling technique and procedures

**Figure 1:**
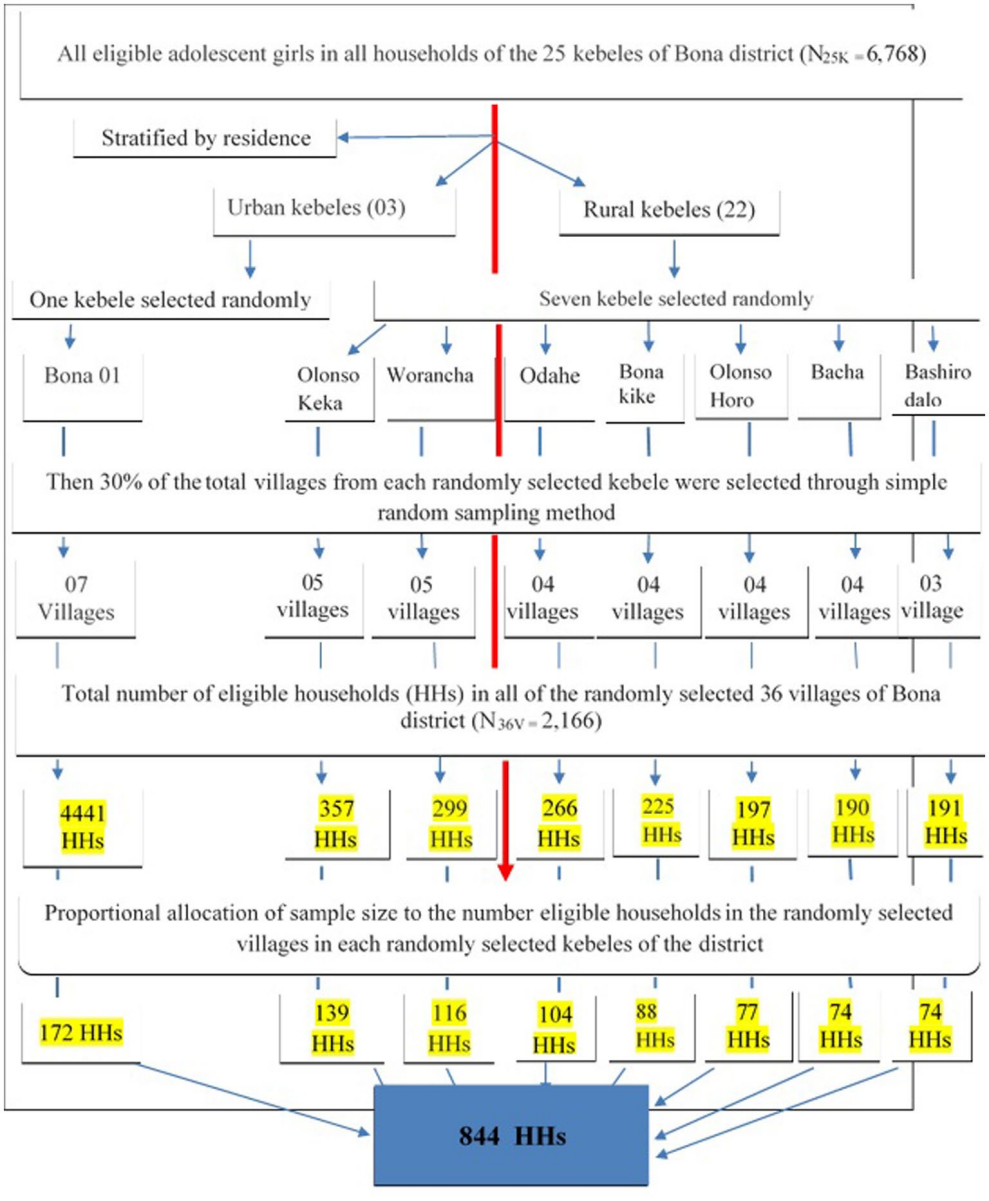
Schematic presentation of sampling procedures for assessment of HPV vaccine uptake and associated factors among adolescent girls aged 14-19 years in Bona district, Sidama region, Ethiopia, 2024 (*n*=844).

A multistage sampling approach was employed to reach the study participants in the district. In the first stage, 25 kebeles of the districts were stratified by their residence status into urban and rural kebeles (3 urban and 22 rural kebeles). Then, by considering the rule of thumb, 30% of the total kebeles in each stratum, one urban and seven rural kebeles were selected by using a simple random sampling technique. Within each randomly selected kebele, again, 30% of the total villages were selected randomly by taking the name and list of all villages as a sampling frame. Then, the sample size was allocated proportionally to the size of households with eligible adolescent girls for each randomly selected village in the kebele. After the determination of the sampling interval (K^th^ value = 2), the study households were systematically selected after selecting the first household randomly and continued every second until the allocated sample size for the villages was obtained. Finally, the selected households were visited for data collection. If two and above eligible adolescent girls were found in the household simple random sampling method was used to select one of them (Figure 1).

### Data collection tools and procedures

Data were collected by using pre-tested, structured and interviewer-administered questionnaires. The tool was adapted after reviewing a previous research that was carried out on relatively the same topic (17–19) and the questionnaire was further evaluated by experts in the area to review its contents. The data collection tool is composed of nine (9) socio-demographic-related questions, twenty-two (22) knowledge-related questions, five (5) attitude-related questions, three (3) HPV vaccine uptake-related questions, and four (4) other essential influencing questions. Under the knowledge-related question, twenty-one total questions (items) were used to assess the knowledge about the HPV vaccine, HPV infection and cervical cancer. The knowledge-related questions were “yes” or “no” type of questions. The questions were partitioned into three sections: Section One: includes seven knowledge-related questions on cervical cancer, Section Two: includes seven knowledge-related questions on HPV infection and Section three includes another seven knowledge questions related to the HPV vaccine.

Participant’s attitude was assessed by using five attitude-related items. Each item was a five-point Likert scale arrangement which included strongly disagree (1), disagree (2), neutral (3), agree (4) and strongly agree (5) were used to assess the overall attitude of the respondents towards HPV vaccine. The internal consistency of the items was tested by using Cronbach’s Alpha to secure the reliability of the items (*α* = 0.86).

### Modality on how to trace study participants

As a procedure, first, the number of all eligible adolescent girls in the randomly selected 36 villages located in the eight randomly selected kebeles were identified and taken from both Health extension workers (Health post) of each kebele and Health Development Army (HDA) leader of the respective village. Then sampling frame for each randomly selected village was adjusted by taking the name and list of all villages as a sampling frame by preparing a unique code number for all eligible households in each randomly selected village separately. Eight health professionals (BSc) who had experience of similar data collection were recruited as data collectors. Another two health professionals (MSc) who had experience in data collection and supervising data collection programs were also assigned as supervisors.

### Data quality control

Language expert translated the questionnaire already prepared in English, into Sidamu Afoo and then it was translated back into English for analysis by the principal investigator to ensure consistency. To assess the face validity of the instrument, the clarity of the questions, and the response of the respondents to the questions, a pretest was done on 5% of the research population in a nearby setting outside the source population; adjustments and modifications were done on the questionnaire based on the results of pretest. Moreover, one-day intensive training was given to data collectors and supervisors by the principal investigator to have a common understanding of the aim of the study, techniques of data collection, procedures to be followed, and contents of the tool and the way how to interview respondents. Data completeness, consistency and accuracy were checked by the data collectors, supervisors and principal investigator every day. The principal investigator and supervisors carried out on-site checks and reviews of all completed questionnaires.

### Operational definition

#### HPV vaccine uptake

In the current study, adolescents who had received at least one dose of HPV vaccine were considered as uptake of HPV vaccine (27).

#### Knowledge

Twenty-one knowledge-related questions (items) were used to assess the knowledge about the HPV vaccine, HPV infection, and cervical cancer. The mean value was used to determine the knowlged level of the participants. Accordingly, those respondents who had scored greater than or equal to the mean value were considered as having good knowledge, whereas those respondents who had scored less than the mean value were considered as having poor knowledge about it (24).

#### Attitude about HPV vaccination

A total of five attitude-related questions were determined by five-point Likert scale items. The mean value was used to determine the attitude level of the participants. Accordingly, those respondents who had scored greater than or equal to the mean value were considered as having good attitude, whereas those respondents who had scored less than the mean value were considered as having poor attitude about it (19).

### Data processing and analysis

After collection, data were checked for completeness and consistency before data entry and cleaning. Then data were entered into Epidata version 4.6 software and exported to the statistical software package for social science (SPSS) version 25.0 for statistical analysis. Data cleaning, refining and recording were done via SPSS version 25.0 software before statistical analysis. Descriptive statistics such as frequencies and percentages were used to describe the results of categorical variables while median and interquartile range were used to describe the results of the continuous variables. Brief texts, tables, and graphs were used to present the results.

A binary logistic regression model was fitted to identify factors associated with HPV vaccine uptake. Candidate variables with *p*-value < 0.25 in bi-variable binary logistic regression were selected to the multivariable binary logistic regression model. Before the inclusion of factors to the final multivariable binary logistic regression model, multicollinearity between independent variables was checked using variance inflation factors (VIFs) and there was no significant multicollinearity (maximum VIF value = 1.28 and mean VIF value = 1.18) for the model. Hosmer Lemeshow goodness of test was also checked to assess the hypothesis of the model fitness and there was no violation of the null hypothesis (*p*-value = 0.21). Then multivariable binary logistic regression was done to control potential confounders and to identify independent factors of HPV vaccine uptake. Both crude (COR) and adjusted odds ratio (AOR) were determined with 95% CI and finally variables with *p*-value <0.05 were declared as factors significantly associated with HPV vaccine uptake during multivariable binary logistic regression.

### Ethical consideration

An ethical clearance letter with the reference number HFE-IRERC-022-2024 was obtained from Hamlin Fistula Ethiopia. Then an official letter of support was also sent to the Bona district health office and a permission letter was also obtained from the authorities in the Bona district health office. Informed consent was obtained from each respondent aged 18 and above years after explaining the purpose and procedure of the study. Written informed consent was obtained separately from parents to ensure their legal preparation on behalf of their children for adolescents aged below 18 years. Participation was voluntary and withdrawal from the study at any time of data collection was considered. No name or other identifying information was included in the questionnaire.

## Results

### Socio-demographic characteristics of the participants

A total of 833 adolescent girls participated in the study, yielding a response rate of 98.7%. The mean age of the participants was 17.14 ± 1.34 years. Most of the participants, 784 (94.1%) were attending school while 49 (5.9%) of them had dropped out of school. The majority, 662 (79.5%) of the participants were rural dwellers and more than one-third of the participant’s mothers (328, 39.4%) had primary (1–8 grade) level educational status. The mean family member size of the households included in the study was 4.72 ± 1.457 SD and more than two-thirds (66.7%) of them had ≤5 family members. In most of the eligible households, 762 (91.5%) had only one eligible adolescent girl (Table 1).

**Table 1:** Socio-demographic characteristics of adolescent girls aged 14–19 years in Bona district, Sidama regional state, Ethiopia, 2024 (*n* = 833).

Variables	Categories	Frequency	Percentage
Age in years	15	138	16.6
	16	145	17.4
	17	163	19.6
	18	239	28.7
	19	148	17.8
Attending school	Yes	784	94.1
	No	49	5.9
Residence	Urban	171	20.5
	Rural	662	79.5
Mother’s educational status	No formal education	118	14.2
	Primary (1–8)	328	39.4
	Secondary (9–12)	260	31.2
	College and above	127	15.2
Father’s educational status	No formal education	91	10.9
	Primary (1–8)	307	36.9
	Secondary (9–12)	270	32.4
	College and above	165	19.8
Occupation of the mother	Housewife	380	45.6
	Merchant	202	24.2
	Government employee	127	15.2
	Non-government employee	84	10.1
	Others*	40	4.8
Occupation of father	Farmer	289	34.7
	Merchant	199	23.9
	Government employee	147	17.6
	Daily laborer	107	12.8
	Non-government employee	53	6.4
	Others*	38	4.6
Family size	≤5	556	66.7
	>5	277	33.3
Number of daughters aged 15–19 years in the family	One	762	91.5
	Two	71	8.5

Others*: privet worker, student, car driver, religious leader.

### Overall knowledge-related characteristics of participants about HPV infection HPV vaccination and cervical cancer

**Figure 2:**
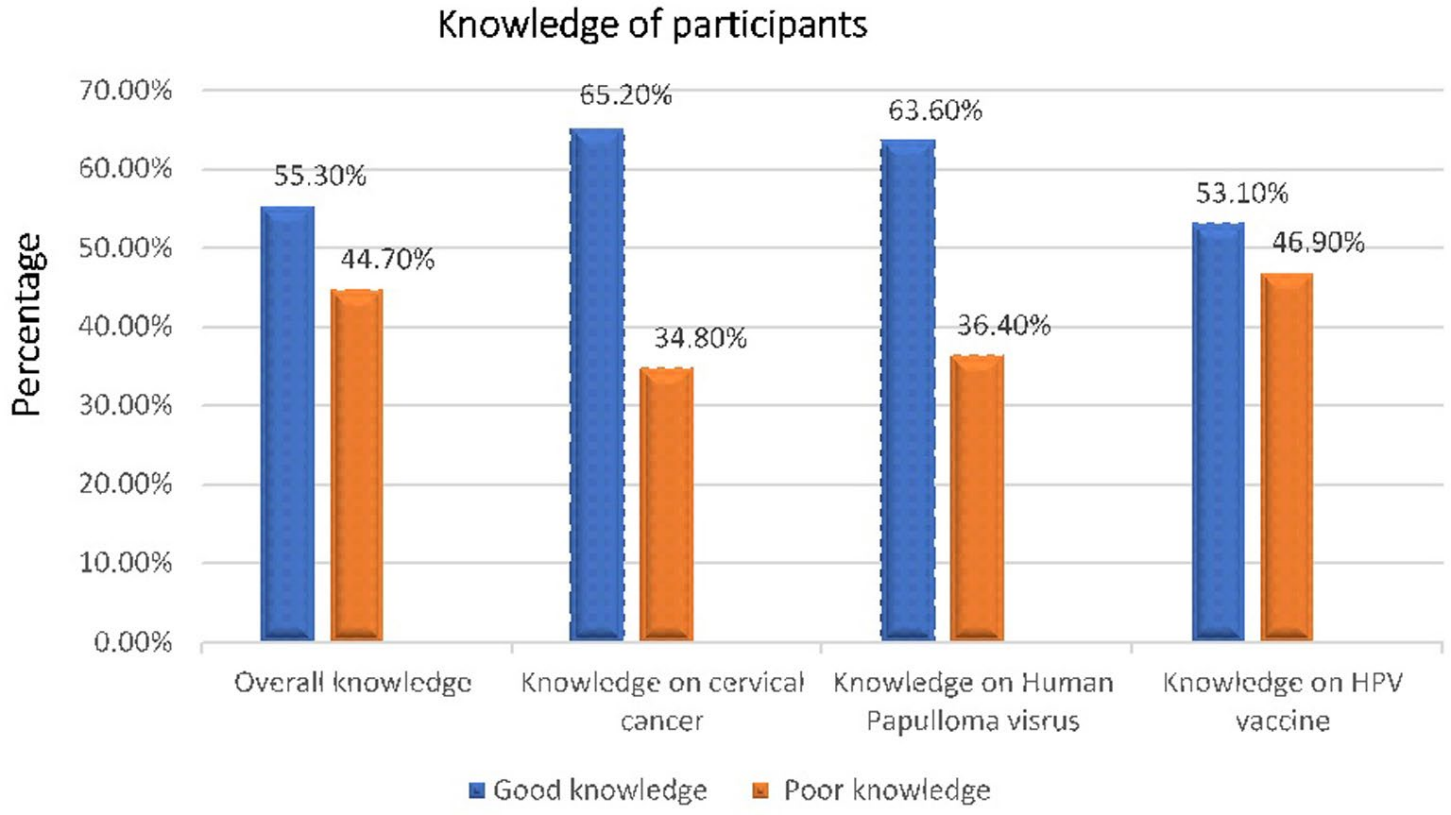
An overall knowledge of adolescent girls aged 14 19 years in Bona distlict, Sidama regional state, Ethiopia, 2024 (*n* = 833).

The study found that 461[55.3%, (95% CI, 52.4–59.7)] of the respondents had an overall good knowledge regarding cervical cancer, HPV infection, and HPV vaccine and 442 (53.1%) of the participants had an overall good regarding HPV vaccine specifically (Figure 2).

### Source of information about HPV

**Figure 3:**
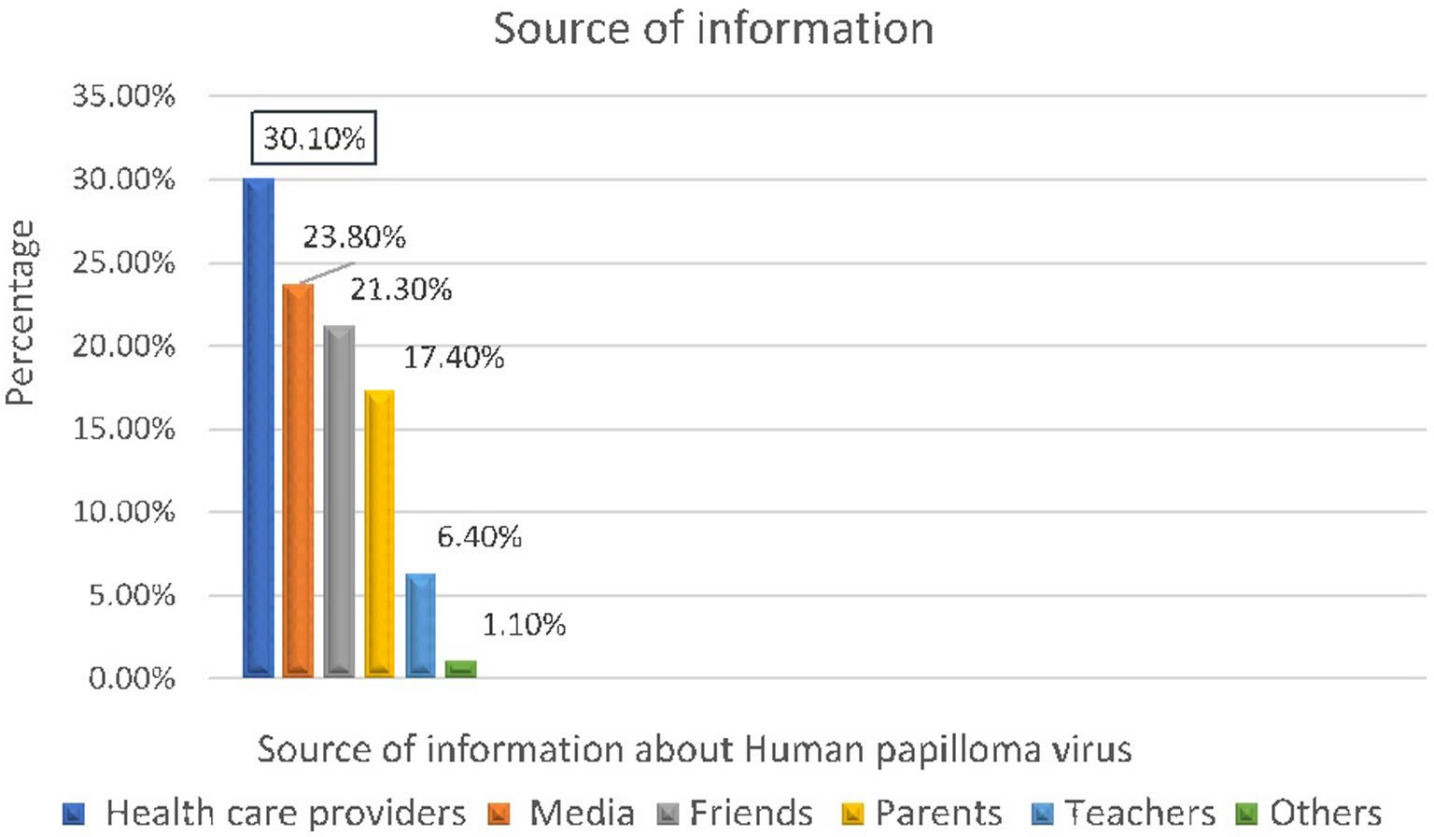
Source of information about human papillomavims of adolescent girls aged 14 19 years in Bona district, Sidama regional state, Ethiopia, 2024 (*n* = 362).

Three hundred sixty-two (43.5%) of the participants had heard about the human papillomavirus. Of the participants who had heard of HPV, 109(13.1%) of them heard from healthcare providers and 86(10.6%) from the media (Figure 3).

### Knowledge about HPV infection, vaccine and cervical cancer

More than two-thirds of the participants, 563 (67.6%) have ever heard about the disease called cervical cancer while 456(54.7%) of them understand cervical cancer is a common cancer in women. Additionally, 388(46.6%) of the adolescent girls knew that all women are at risk of developing cervical cancer and 498 (59.8%) of them were aware that cervical cancer can be caused by sexually transmitted diseases. The majority of the respondents, 603 (72.4%), reported that having multiple sexual partners increases the risk of HPV infection and 416 (49.9%) of them noted that people can get HPV infection for a long time without knowing it. Of the adolescent girls, 522 (62.7%) knew that HPV infection has a vaccine and 490 (58.8%) of them understood that HPV vaccination effectively prevents cervical cancer (Table 2).

**Table 2:** Specific knowledge-related about HPV, vaccination, cervical cancer among adolescent girls aged 14–19 years in Bona district, Sidama regional state, Ethiopia, 2024 (*n* = 833).

Variables	Categories	Frequency	Percentage
Cervical cancer-related questions			
Have ever heard about cervical cancer	Yes	563	67.6
	No	270	32.4
Cervical cancer is a common cancer in women	Yes	456	54.7
	No	377	45.3
All women are at risk of developing cervical cancer	Yes	388	46.6
	No	445	53.4
Cervical cancer can be caused by sexually transmitted disease	Yes	498	59.8
	No	335	40.2
Symptoms of cervical cancer could not be recognized at an early stage	Yes	414	49.7
	No	419	50.3
Cervical cancer is preventable	Yes	595	71.4
	No	238	28.6
Early-stage cervical cancer is treatable	Yes	331	39.7
	No	502	60.3
Human papillomavirus related questions			
Have you ever heard about human papilloma virus?	Yes	362	43.5
	No	471	56.5
HPV causes cervical cancer	Yes	358	43
	No	475	57
HPV infection is a sexually transmitted infection	Yes	494	59.3
	No	339	40.7
Sex at an early age increases the risk of HPV infection	Yes	427	51.3
	No	406	48.7
Having multiple sexual partners increases the risk of HPV infection	Yes	603	72.4
	No	230	27.6
People can get HPV infection for a long time without knowing it	Yes	416	49.9
	No	417	50.1
HPV virus can be cleared from the body Without treatment in some individuals	Yes	545	65.4
	No	288	34.6
HPV vaccination related questions			
Do you know HPV infection has a vaccine?	Yes	522	62.7
	No	311	37.3
HPV vaccination effectively prevents cervical cancer	Yes	490	58.8
	No	343	41.2
Screening for cervical cancer is necessary after receiving the HPV vaccination	Yes	446	53.5
	No	387	46.5
HPV vaccine should be given before the first sexual intercourse	Yes	379	45.5
	No	454	54.5
HPV vaccine can be offered to female children greater than or equal to 9 years of age	Yes	331	39.7
	No	502	60.3
Complete HPV vaccination requires two doses	Yes	344	41.3
	No	489	58.7
The HPV vaccine is delivered over a 6 month schedule	Yes	436	52.3
	No	397	47.7

### Attitude towards HPV vaccination

**Figure 4:**
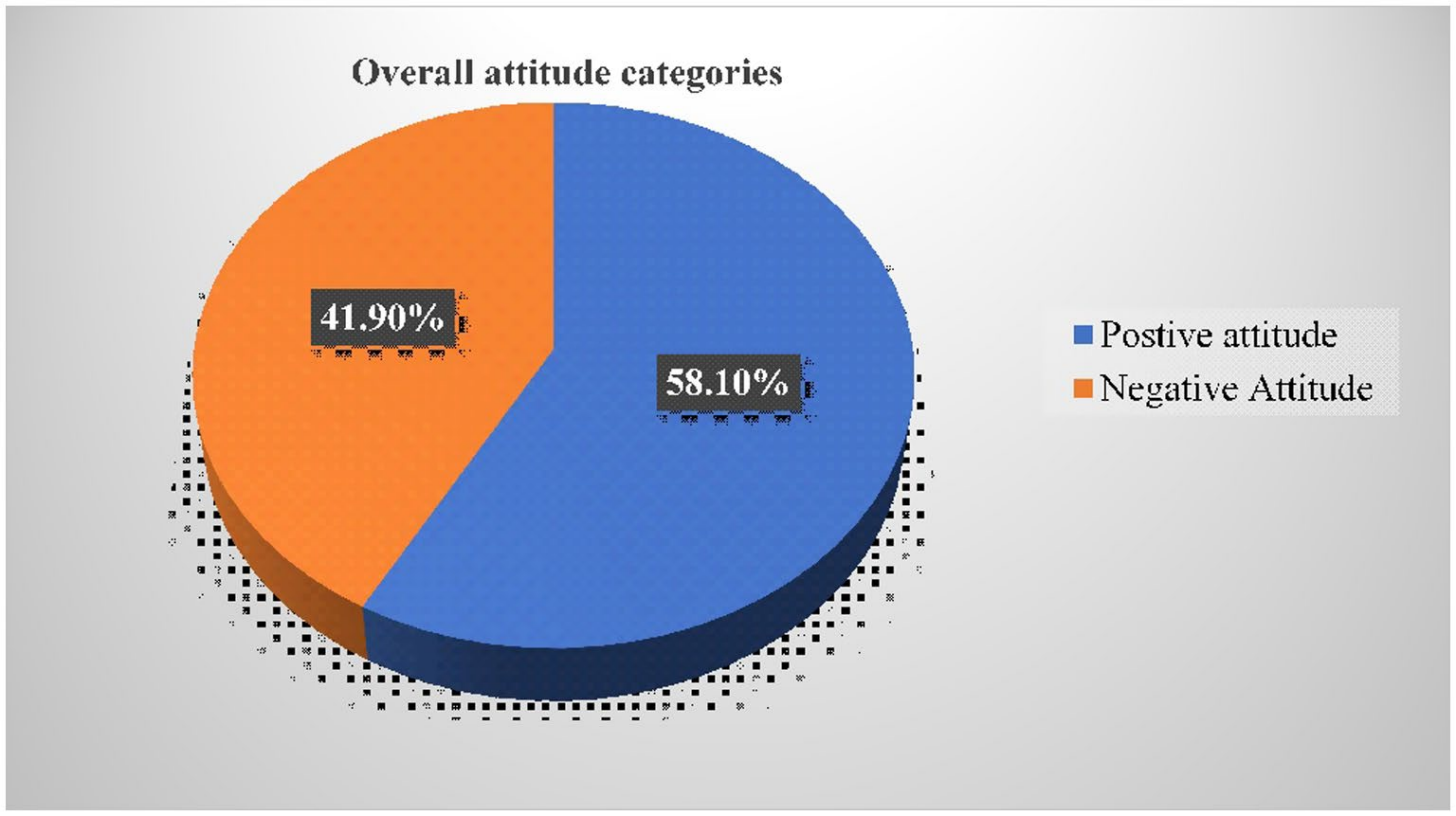
Categories of an overall (cumulative) attitude towards HPV vaccination of adolescent girls aged 14 — 19 years in Bona district, Sidama regional state, Ethiopia, 2024 (*n* = 833).

The study reported that 484 [58.1%, (95% CI: 54.6–62.5)] of participants had an overall positive attitude towards HPV vaccination (Figure 4).

Of the respondents, 95(11.4%) strongly disagreed that if they feel at risk of getting HPV, they would take the vaccine while 190 (22.8%) of them strongly agreed that they would take the vaccine if they feel at risk of getting HPV. Additionally, 139(16.7%) of the participants disagreed that taking the vaccine would keep them safe and healthy while 323 (38.8%) of them agreed that taking the vaccine would keep them safe and healthy (Table 3).

**Table 3:** Specific attitude-related items on HPV vaccination of adolescent girls aged 14–19 years in Bona district, Sidama regional state, Ethiopia, 2024 (*n* = 833).

Variables	Categories	Frequency	Percentage
Because I feel at risk of getting HPV, I will take the vaccine	Strongly disagree	95	11.4
	Disagree	112	13.4
	Neutral	138	16.6
	Agree	298	35.8
	Strongly agree	190	22.8
I feel being infected with HPV is very deadly and can lead to death	Strongly disagree	119	14.3
	Disagree	136	16.3
	Neutral	73	8.8
	Agree	300	36
	Strongly agree	205	24.6
I think it is not easy to find a place to receive the HPV vaccination	Strongly disagree	75	9
	Disagree	177	21.2
	Neutral	64	7.7
	Agree	293	35.2
	Strongly agree	224	26.9
I think taking the vaccine will keep me safe and healthy	Strongly disagree	45	5.4
	Disagree	139	16.7
	Neutral	101	12.1
	Agree	323	38.8
	Strongly agree	225	27
I would need the HPV vaccine if I had multiple sexual partners	Strongly disagree	71	8.5
	Disagree	161	19.3
	Neutral	84	10.1
	Agree	327	39.3
	Strongly agree	190	22.8

### Prevalence of HPV vaccine uptake among 14–19 years old adolescent girls

This study found that the prevalence of HPV vaccination uptake among female adolescent girls aged 15–19 years in the study area was, 413 [49.58% (95% CI 46.18–52.98)]. Likewise, the study revealed that of the participants who received HPV vaccination, 217 (52.54%) of them received one HPV vaccine dose and 196 (47.46%) of them received two doses.

### Factors associated with HPV vaccination uptake

The study revealed that the odds of HPV vaccination uptake were about 2.8 times higher among adolescent girls who live in urban areas as compared to rural dwellers [AOR = 2.84(95% CI: 1.87–4.31)]. Similarly, the study showed the odds of HPV vaccination uptake were about 1.8 times more likely among adolescent girls whose mothers have college and above educational status as compared to those with no formal education [AOR = 1.79 (95% CI 1.23–3.67)]. The odds of getting HPV vaccination were also 2.5 times higher among respondents who had good knowledge of cervical cancer, HPV infection, and HPV vaccine than those who had poor knowledge [AOR = 2.53 (1.82–3.51)]. Likewise, the likelihood of HPV vaccine uptake was about two times more likely among respondents who had a positive attitude towards the HPV vaccine as compared to those who had a negative attitude [AOR = 2.12 (95% CI: 1.53–2.94)] (Table 4).

**Table 4:** Multi-variable binary logistic regression analysis of associated factors with HPV vaccination uptake among adolescent girls aged 14–19 years in Bona district, Sidama regional state, Ethiopia, 2024 (*n* = 833).

Variables		HPV vaccine		COR (95% CI)	AOR (95% CI)	*p*-value
		Received *N* (%)	Not received *N* (%)			
Age				0.87 (0.79–0.96)	0.88 (0.78–1.07)	0.054
Residence	Urban	126 (73.7)	45 (26.3)	3.66 (2.52–3.32)	2.84 (1.87–4.31)*	<0.001
	Rural	287 (43.4)	375 (56.6)	1.00 (reference)	1.00 (reference)	
Mothers educational status	No formal	33 (31.4)	85 (68.6)	1.00 (reference)	1.00 (reference)	
	Primary	167 (50.9)	161 (49.1)	1.11 (0.72–1.69)	1.10 (0.70–1.65)	0.06
	Secondary	141 (54.2)	119 (45.8)	1.26 (0.84–1.91)	1.22 (0.86–1.89)	0.052
	College+	72 (56.7)	55 (43.3)	3.37 (1.98–5.78)	1.79 (1.23–3.67)*	0.031
Fathers educational status	No formal	41 (45.1)	50 (54.9)	1.00 (reference)	1.00 (reference)	
	Primary	143 (46.6)	164 (53.4)	1.37 (0.93–2.03)	1.32 (0.94–2.08)	0.29
	Secondary	134 (49.6)	136 (50.4)	1.55 (1.06–2.28)	1.43 (0.96–2.17)	0.56
	College+	85 (54.8)	70 (45.2)	1.65 (0.98–2.77)	1.54 (0.97–2.12)	0.72
Family size	≤5	285 (51.3)	271 (48.7)	1.22 (0.93–1.63)	1.15 (0.8–1.64)	0.456
	>5	128 (46.2)	149 (53.8)	1.00 (reference)	1.00 (reference)	
Knowledge	Good	272 (59)	189 (41)	2.36 (1.78–3.12)	2.53 (1.82–3.51)*	<0.001
	Poor	141 (37.9)	231 (62.1)	1.00 (reference)	1.00 (reference)	
Attitude towards HPV vaccinations	Positive	270 (55.8)	214 (44.2)	1.82 (1.38–2.40)	2.12 (1.53–2.94)*	0.002
	Negative	143 (40.9)	206 (59.1)	1.00 (reference)	1.00 (reference)	

*Adjusted odd ratio not including one.

## Discussion

The study found that the prevalence of HPV vaccination uptake among female adolescent girls aged 14–19 years was 49.58%. The magnitude of HPV vaccination uptake in this study (49.58%) was found to be in line with findings from research conducted in the towns of Nekemte in western Ethiopia (52%) (31), Mettu town in southwest Ethiopia (48.6%) (19), and Gambella town in southwest Ethiopia (48%) (25) Furthermore, the magnitude of HPV vaccination uptake in this study (49.58%) was found to be comparable to research conducted in Malaysia (50.1%) (32) and Indonesia (50.7%) (33). These parallels may result from the research participants’ comparable sociodemographic backgrounds and the HPV vaccine’s consistent availability.

However, the magnitude of HPV vaccine uptake was lower than the findings of studies carried out in Brazil (83.5%) (34) and South Africa (75%) (35). This discrepancy may result from variations in the sociodemographic traits of research participants and research settings among the studies. In contrast to this study, these countries anticipated to have more vaccination accessibility and vaccine awareness, which might boost the rate of HPV vaccine uptake in these areas.

On the other hand, the HPV vaccination uptake rate among adolescent girls in this study (49.58%) was found to be higher than the results of studies conducted in Lira City, northern Uganda (19.6%) (36), Ambo town, central Ethiopia (44.4%) (27), and Bahridar city, northwest Ethiopia (45.3%) (24). These differences may result from different approaches to defining or quantifying HPV vaccine uptake and variations in participant attitudes toward the vaccine. For example, in the Ugandan study, HPV vaccination uptake was defined as receiving three doses of the vaccine according to the recommended schedule; whereas, in this study, individuals who had ever received the HPV vaccine were considered to have received the vaccine. Additionally, about 67.3 and 64.7% of the participants in the studies done in Bahridar city and Ambo town had a negative attitude towards the HPV vaccine, respectively, while around 58% of the respondents in this study had a positive attitude towards it. This could comparatively increase the prevalence of HPV vaccine uptake in this study since the respondents’ attitude status was strongly associated with the magnitude of HPV vaccine uptake in this study.

In terms of the significant factors, the study found that adolescent girls living in urban areas had around three times higher HPV vaccine uptake rates than girls living in rural areas. Research conducted in the Minjar Shenkora area in the north Shoa zone of Ethiopia (26) provided evidence in support of this hypothesis, showing a strong positive correlation between urban residency and HPV vaccine uptake. This could be because urban residents would have better access to information about the benefits of HPV and also get the services at ease without challenges.

According to the study, teenage girls whose moms have a college degree or higher are approximately two times more likely to have had an HPV vaccination than teenage girls whose mothers have never attended school. This finding was in line with research conducted in the towns of Gambella and Mettu (19, 25), which found a favorable correlation between mothers education level and HPV vaccination uptake. By gathering medical knowledge about the dangers of acquiring HPV infections on their own without contacting healthcare practitioners, it is evident that informed moms feel comfortable vaccinating their daughters against the HPV virus (36). In addition, it was discovered that respondents with good knowledge about HPV infection, vaccine, and cervical cancer had a nearly 2.5-fold increased likelihood of receiving the vaccination than did those with poor knowledge. This result was also consistent with research conducted in Nekemte city, Ethiopia (31) and Malaysia (32). One plausible reason is that individuals with a strong understanding of the HPV vaccination contribute to enhancing its advantages, facilitating a positive attitude toward the vaccine, and encouraging teenagers to get the vaccine (26).

Similarly, those who had a good attitude about the HPV vaccine were roughly twice as likely to have taken it as those who had a negative one. The findings were corroborated by research conducted in Indonesia (33) and Gambella town (19). This makes sense because vaccination is a recommended practice and is reinforced by good sentiments regarding HPV vaccination. Teenagers’ good attitudes, then, are the driving forces behind their practice, and this leads to the conclusion that attitude influences behavior (31).

### Strengths and limitations of the study

The use of a relatively representative sample size and employing a community-based study design, supported by a thorough assessment tool, successfully identified the factors influencing HPV vaccination uptake among adolescent girls in the district and accurately reflected actual uptake of the HPV vaccination. However, the possibility of social desirability bias and recall bias might not be eliminated despite the great efforts made, such as the crosschecking of the vaccination card and granting anonymity and total confidentiality to reduce social desirability bias.

### Conclusions and recommendations

The study found that the prevalence of HPV vaccination uptake among female adolescent girls was low in the study area compared to international targets. Being an urban resident, mother’s educational level with college and above, the participant’s good knowledge of cervical cancer, human papillomavirus, and the HPV vaccine, as well as having a towards the HPV vaccine, were positively associated with HPV vaccination uptake in the study area. The study implies that empowering women through education, promoting health awareness about HPV, cervical cancer, and the HPV vaccine, and implementing targeted interventions for rural populations are essential means to increase HPV vaccine uptake.

## Data Availability

The raw data supporting the conclusions of this article will be made available by the authors, without undue reservation.

## References

[1] Bosch FX Lorincz A Muñoz N Meijer CJ Shah KV. The causal relation between human papillomavirus and cervical cancer. J Clin Pathol. (2002) 55:244–65. doi: 10.1136/jcp.55.4.244, PMID: 11919208 PMC1769629

[2] Cheng L Wang Y Du JJV. Human papillomavirus vaccines: an updated review. Vaccine. (2020) 8:391. doi: 10.3390/vaccines8030391, PMID: 32708759 PMC7565290

[3] Berman TA Schiller JTJC. Human papillomavirus in cervical cancer and oropharyngeal cancer: one cause, two diseases. Cancer. (2017) 123:2219–29. doi: 10.1002/cncr.30588, PMID: 28346680

[4] Okunade KS. Human papillomavirus and cervical cancer. J Obstet Gynaecol. (2020) 40:602–8. doi: 10.1080/01443615.2019.1634030, PMID: 31500479 PMC7062568

[5] World Health Organization. Global strategy to accelerate the elimination of cervical cancer as a public health problem. Geneva: World Health Organization (2020).

[6] World Health Organization. (2024). Cervical Cancer. Available online at: https://www.who.int/news-room/fact-sheets/detail/cervical-cancer?gad_source=1&gclid=CjwKCAjwlbu2BhA3EiwA3yXyu9TO1vxhAXTCQyPG3jU797l75mSPBBRDu0R9qJn_8WYQBlxlzM6NjxoCESkQAvD_BwE (Accessed August 28, 2024).

[7] Gray A Fisher CBJFiPH. Factors associated with HPV vaccine acceptability and hesitancy among Black mothers with young daughters in the United States. Front Public Health. (2023) 11:1124206. doi: 10.3389/fpubh.2023.1124206, PMID: 37139381 PMC10150885

[8] Bruni L Albero G Serrano B Mena M Collado JJ Gómez D. (2019). Human papillomavirus and related diseases report. 7: 26–42. 10.1016/j.pvr.2018.12.002 PMC6329707 30599280

[9] Chauke-Moagi BE Mumba M. New vaccine introduction in the East and Southern African sub-region of the WHO African region in the context of GIVS and MDGs. Vaccine. (2012) 30:C3–8. doi: 10.1016/j.vaccine.2012.05.086, PMID: 22939018

[10] Arbyn M Xu L Simoens C Martin-Hirsch PP. Prophylactic vaccination against human papillomaviruses to prevent cervical cancer and its precursors. Cochrane Database Syst Rev. (2018) 5:Cd009069. doi: 10.1002/14651858.CD009069.pub3, PMID: 29740819 PMC6494566

[11] Brisson M Kim JJ Canfell K Drolet M Gingras G Burger EA. Impact of HPV vaccination and cervical screening on cervical cancer elimination: a comparative modelling analysis in 78 low-income and lower-middle-income countries. Lancet. (2020) 395:575–90. doi: 10.1016/S0140-6736(20)30068-4, PMID: 32007141 PMC7043009

[12] World Health Organization. Considerations for human papillomavirus (HPV) vaccine product choice. Geneva, Switzerland: World Health Organization (2024).

[13] Asgedom YS Kebede TM Seifu BL Mare KU Asmare ZA Asebe HA. Human papillomavirus vaccination uptake and determinant factors among adolescent schoolgirls in sub-Saharan Africa: A systematic review and meta-analysis. Hum Vaccin Immunother. (2024) 20:2326295. doi: 10.1080/21645515.2024.2326295, PMID: 38505959 PMC10956624

[14] Asempah E Ikpebe E. Accelerating HPV vaccination in Africa for health equity. Glob Health Res Policy. (2024) 9:37. doi: 10.1186/s41256-024-00380-z, PMID: 39294815 PMC11411759

[15] Kurosawa M Sekine M Yamaguchi M Kudo R Hanley SJB Hara M. Long-term effects of human papillomavirus vaccination in clinical trials and real-world data: a systematic review. Vaccine. (2022) 10:256. doi: 10.3390/vaccines10020256, PMID: 35214713 PMC8877934

[16] Rimer B.J. (2018). HPV vaccination for cancer prevention: progress, opportunities, and a renewed call to action.

[17] Bruni L Saura-Lázaro A Montoliu A Brotons M Alemany L Diallo MS. HPV vaccination introduction worldwide and WHO and UNICEF estimates of national HPV immunization coverage 2010–2019. Prev Med. (2021) 144:106399. doi: 10.1016/j.ypmed.2020.106399, PMID: 33388322

[18] World Health Organization. Meeting of the strategic advisory group of experts on immunization, October 2014—conclusions and recommendations. Wkly Epidemiol Rec. (2014) 89:561–76. PMID: 25513671

[19] Dawud A Kera AM Bekele D Hiko D Zewdie A. Factors associated with uptake of human papillomavirus vaccination among adolescent girls in Mettu town, southwest Ethiopia: a school-based cross-sectional study. BMJ Open. (2023) 13:e071878. doi: 10.1136/bmjopen-2023-071878, PMID: 37996240 PMC10668246

[20] World Health Organization. (2018). Ethiopia launches human papillomavirus vaccine for 14 year old girls. Available online at: https://www.afro.who.int/news/ethiopia-launches-human-papillomavirus-vaccine-14-year-old-girls#:~:text=Addis%20Ababa%2C%20Ethiopia%203rd%20December,are%2014%20years%20of%20age (Accessed 2024).

[21] Alene T Atnafu A Mekonnen ZA Minyihun A. Acceptance of human papillomavirus vaccination and associated factors among parents of daughters in Gondar town, Northwest Ethiopia. Cancer Manag Res. (2020) 12:8519–26. doi: 10.2147/CMAR.S275038, PMID: 32982444 PMC7502398

[22] Destaw A Yosef T Bogale B. Parents willingness to vaccinate their daughter against human papilloma virus and its associated factors in Bench-Sheko zone, southwest Ethiopia. Heliyon. (2021) 7:e07051. doi: 10.1016/j.heliyon.2021.e07051, PMID: 34041397 PMC8141465

[23] World Health Organization. (2019). Accelerating the elimination of cervical cancer as a global public health problem. World Health Organization. Regional Office for South-East Asia.

[24] Lakneh EA Mersha EA Asresie MB Belay HG. Knowledge, attitude, and uptake of human papilloma virus vaccine and associated factors among female preparatory school students in Bahir Dar City, Amhara Region, Ethiopia. PLoS One. (2022) 17:e0276465. doi: 10.1371/journal.pone.0276465, PMID: 36409675 PMC9678319

[25] Woldehawaryat EG Geremew AB Asmamaw DBJBo. Uptake of human papillomavirus vaccination and its associated factors among adolescents in Gambella town, Southwest, Ethiopia: a community-based cross-sectional study. BMJ Open. (2023) 13:e068441. doi: 10.1136/bmjopen-2022-068441, PMID: 37669848 PMC10481830

[26] Kassa HN Bilchut AH Mekuria AD Lewetie EM. Practice and associated factors of human papillomavirus vaccination among primary school students in Minjar-Shenkora district, North Shoa Zone, Amhara Regional State, Ethiopia, 2020. Cancer Manag Res. (2021) 13:6999–7008. doi: 10.2147/CMAR.S324078, PMID: 34522142 PMC8434827

[27] Beyen MWm Bulto GA Chaka EE Debelo BT Roga EY Wakgari N. Human papillomavirus vaccination uptake and its associated factors among adolescent school girls in Ambo town, Oromia region, Ethiopia, 2020. PLoS One. (2022) 17:e0271237. doi: 10.1371/journal.pone.0271237, PMID: 35830389 PMC9278730

[28] Larebo YM Elilo LT Abame DE Akiso DE Bawore SG Anshebo AA. Awareness, acceptance, and associated factors of human papillomavirus vaccine among parents of daughters in Hadiya Zone, Southern Ethiopia: a cross-sectional study. Vaccine. (2022) 10:1988. doi: 10.3390/vaccines10121988, PMID: 36560398 PMC9785952

[29] Agimas MC Adugna DG Derseh NM Kassaw A Kassie YT Abate HK. Uptake of human papilloma virus vaccine and its determinants among females in East Africa: a systematic review and meta-analysis. BMC Public Health. (2024) 24:842. doi: 10.1186/s12889-024-18141-5, PMID: 38500046 PMC10949808

[30] World Health Organization, W.H.O.J.G.W.H. (2021). Human papillomavirus (HPV) vaccination coverage.

[31] Abera M. (2023). Human papillomavirus vaccination practice and its associated factors among secondary school female students in Nekemte town, Oromia region, Ethiopia, 2022

[32] Al-Naggar RA Bobryshev YV Al-Jashamy K Al-Musli M. Practice of HPV vaccine and associated factors among school girls in Melaka, Malaysia. Asian Pac J Cancer Prev. (2012) 13:3835–40. doi: 10.7314/APJCP.2012.13.8.3835, PMID: 23098480

[33] Aimagambetova G Babi A Issa T Issanov A. What factors are associated with attitudes towards HPV vaccination among Kazakhstani women? Exploratory analysis of cross-sectional survey data. Vaccine. (2022) 10:824. doi: 10.3390/vaccines10050824, PMID: 35632580 PMC9146459

[34] Williams WW Lu PJ Saraiya M Yankey D Dorell C Rodriguez JL. Factors associated with human papillomavirus vaccination among young adult women in the United States. Vaccine. (2013) 31:2937–46. doi: 10.1016/j.vaccine.2013.04.041, PMID: 23643629 PMC8972189

[35] Dike SNJNJ. Factors associated with African American mothers’ perceptions of human papillomavirus vaccination of their daughters: an integrated literature review. Oncol Nurs Forum. (2021) 48:371–89. doi: 10.1188/21.ONF.371-389, PMID: 34142996

[36] Nakayita RM Benyumiza D Nekesa C Misuk I Kyeswa J Nalubuuka A. Factors associated with uptake of human papilloma virus vaccine among school girls aged 9-14 years in Lira City northern Uganda: a cross-sectional study. BMC Womens Health. (2023) 23:362. doi: 10.1186/s12905-023-02511-z, PMID: 37420225 PMC10329291

